# circRNA expression profiling of colon tissue from mesalazine-treated mouse of inflammatory bowel disease reveals an important circRNA-miRNA-mRNA pathway

**DOI:** 10.18632/aging.202780

**Published:** 2021-03-26

**Authors:** Wei Zhou, Haiyin Zhang, Yibin Pan, Yanwu Xu, Yongqing Cao

**Affiliations:** 1 Department of Anal-Rectal Surgery, Longhua Hospital, Shanghai University of Traditional Chinese Medicine, Shanghai, China; 2 Department of Pharmacology, Yale University School of Medicine, New Haven, Connecticut 06510, United States of America; 3 Department of Biochemistry, School of Basic Medicine, Shanghai University of Traditional Chinese Medicine, Shanghai, China

**Keywords:** circRNA, inflammatory bowel disease, mesalazine, dextran sulfate sodium, RNA-seq

## Abstract

Mesalazine (5-aminosalicylic acid, 5-ASA) has been widely used to treat inflammatory bowel disease (IBD). However, it remains unclear about the underlying biological mechanisms of IBD pathogenesis and mesalazine treatment, which could be partially clarified by exploring the profiling of circular RNAs (circRNAs) using RNA-seq. A total of 15 mice (C57BL/6) were randomly assigned to three equally sized groups: control, dextran sulfate sodium (DSS, using DSS to induce IBD), and DSS+5-ASA (using mesalazine to treat IBD). We randomly selected three mice of each group to collect colon tissues for RNA-seq and then performed bioinformatic analysis for two comparisons: DSS vs. control and DSS+5-ASA vs. DSS. Comparisons of a series of indicators (e.g., body weight) verified the establishment of DSS-induced IBD mouse model and the effectiveness of mesalazine in treating IBD. We identified 182 differentially expressed circRNAs, including 55 up-regulated and 47 down-regulated circRNAs when comparing the DSS+5-ASA with the DSS group. These 102 circRNA-associated genes were significantly involved in the N-Glycan biosynthesis and lysine degradation. The network analysis of circRNA-miRNA-mRNAs identified an important pathway, i.e., chr10:115386962-115390436+/mmu-miR-6914-5p/Atg7, which is related to autophagy. The findings provide new insights into the biological mechanisms of IBD pathogenesis and mesalazine treatment, particularly highlighting the circRNA-miRNA-mRNA pathway.

## INTRODUCTION

Inflammation has been involved in many chronic diseases including aging symptoms. Inflammatory bowel disease (IBD) is an idiopathic inflammatory disorder of gastrointestinal tract, including Crohn’s disease (CD) and ulcerative colitis (UC). Over the past 20 years, the incidence and prevalence of IBD have soared in developing countries in Asia (especially in East Asia), South America, the Middle East and Africa. The total number of IBD cases in China from 2005 to 2014 is about 350,000, and it is estimated that Chinese patients will reach to 1.5 million by 2025 [1]. Although European and American patients account for only 0.5% of the total number of IBD cases worldwide, IBD is increasingly influencing the general population worldwide [2]. Genetic and environmental factors are generally considered to be related to the occurrence of IBD; whereas the detailed etiology and pathogenesis of IBD remain elusive [3]. It has been demonstrated that the response of the intestinal mucosal immune system to some abnormal circumstances may cause inflammation, leading to the development of IBD [4]. Correspondingly, some studies reported that mesalazine (5-aminosalicylic acid, 5-ASA), which has been widely used to treat IBD in clinical settings [5], may play a therapeutic role by reducing the expression levels of pro-inflammatory cytokines including interleukin 6 (IL-6) and tumor necrosis factor alpha (TNF-α) in colon tissues [6, 7]. As a treatment drug for IBD, mesalazine has advantages such as high safety and few adverse reactions. However, it remains unclear about the molecular mechanism of mesalazine in IBD therapeutic process.

Circular RNAs (circRNAs) are the novel star molecule, which forms covalent closed loops with different sizes and sources, and represent a class of abundant, stable and widely occurring RNA molecules in animals. As endogenous noncoding RNAs, circRNAs are more stable than linear RNAs, because of their closed loop structures [8, 9]. They play an important role in biological processes. For example, as a miRNA sponge [10], RNA-binding proteins, mRNA "magnets" to guide protein translation, and serve as a biomarker for diagnosis and prognosis of some diseases, such as cancers [11]. Compared with the classical transcripts, circRNAs are characterized by circular splicing. The complete identification of splice reads facilitates the accurate assessment of the abundance of circRNA. Generally, the number of spliced reads is used to measure the expression level of circRNA. Currently, a few studies have reported the functions of circRNAs in IBD patients and colitis-induced colon carcinoma mice [12, 13]. However, to the best of our knowledge, no studies have used RNA-seq to comprehensively explore the expression profiling of circRNAs when using mesalazine to treat IBD, in either human or animal colon tissues.

**Figure 1:**
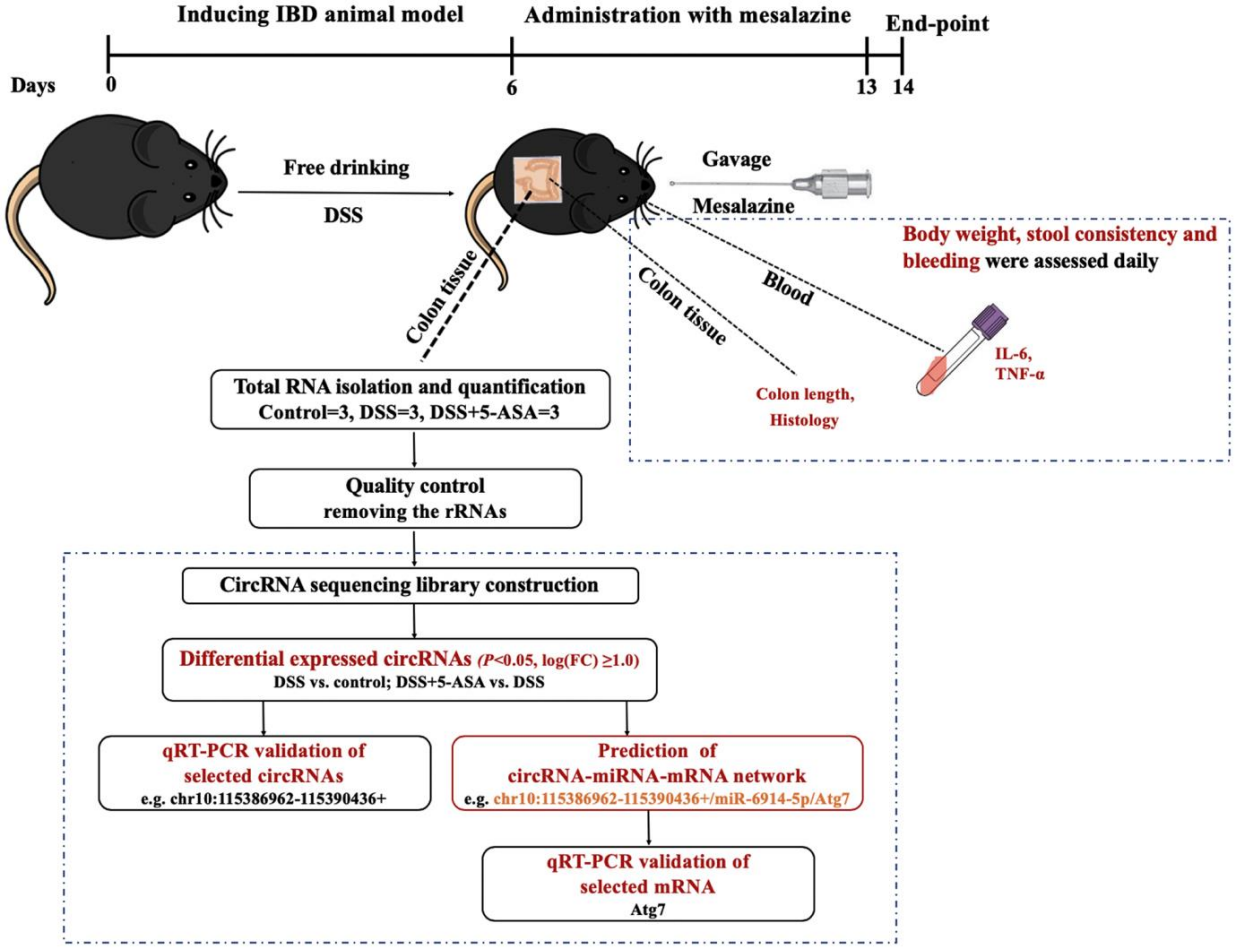
**The workflow of this study.** We first induced IBD mouse model with DSS, and then administrated mesalazine by gavage. At the end of the experiment, we collected colon tissues and blood from eyes of mice to measure colon length, conducted H&E staining, and determined the expression levels of inflammation-related cytokines IL-6 and TNF-α in serum. At the same time, we explored the circRNA expression profiling of colon tissue using next-generation RNA-seq and then carried out a series of bioinformatics analysis and preliminary validation experiments.

In this study, we first demonstrated that an IBD mouse model was successfully established using dextran sulfate sodium (DSS) by assessing a series of indicators (e.g., body weight, colon length, expression levels of inflammatory cytokines), and then administrated mesalazine and observed significant changes (e.g., attenuated colonic inflammation) in these indicators. We then used next-generation RNA-seq to explore the expression profiling and potential roles of circRNAs in the colonic inflammation of DSS-induced and mesalazine-treated mice based on the established mouse model. We also used qRT-PCR to further validate the differentially expressed circRNAs. We found an underlying pathway of circRNA-miRNA-mRNA relevant to the therapeutic process of mesalazine for DSS-induced IBD, suggesting the potentials of the circRNAs serving as new biomarkers for IBD progression. The workflow of this study is shown in Figure 1.

## RESULTS

### DSS induced IBD and mesalazine attenuated colonic inflammation

As generally done in literature, changes in body weight, colon length and disease activity index (DAI) [14] were analyzed and the serum levels of IL-6 and TNF-α were measured. As shown in Supplementary Figure 1A, mice treated with 2.5% DSS alone exhibited progressive weight loss compared to mice in control group since Day 5, whereas mice that received mesalazine were lower than that of control group since Day 7, the weight of DSS-administration mice decreased rapidly from the Day 4 to Day 9, while the mice of DSS+5-ASA group began to lose weight from the Day 5 to Day 9. Beginning on the Day 9, the weight of the two groups tended to be stable. DAI results showed the scores of each group were 0 in the first four days, the scores for DSS group and DSS+5-ASA group began to rise on Day 5, and from then on, the score for DSS group was significantly higher than that of the other two groups and began to decrease on Day 10 and then tended to be stable. The score for DSS+5-ASA group was significantly higher on Day 7 and began to decrease on Day 12. There was no significant difference between DSS and DSS+5-ASA group (Supplementary Figure 1B). Moreover, the average colon length of control group, DSS group and DSS+5-ASA group was 9.4 cm, 6.7 cm and 8.5 cm, respectively. Shortening of the colon was clearly observed in mice with DSS-induced IBD. These changes were significantly ameliorated when using mesalazine treatment (Supplementary Figure 1C). Histology results confirmed the clinical signs of IBD by demonstrating the characteristic pattern of inflammation, including reactive epithelial atypia and architectural distortion. The tissue damage was attenuated in mice from DSS+5-ASA group (Supplementary Figure 1D). The serum expression levels of IL-6 and TNF-α were significantly increased in the DSS group compared with the control group, which were decreased in the DSS+5-ASA group compared with the DSS group (Supplementary Figure 1E, 1F). These results indicate that we had successfully established the IBD mouse model using DSS and mesalazine played a protective role during the progression of IBD.

### Expression profiling of circRNA in DSS-induced and mesalazine-treated IBD model

**Figure 2:**
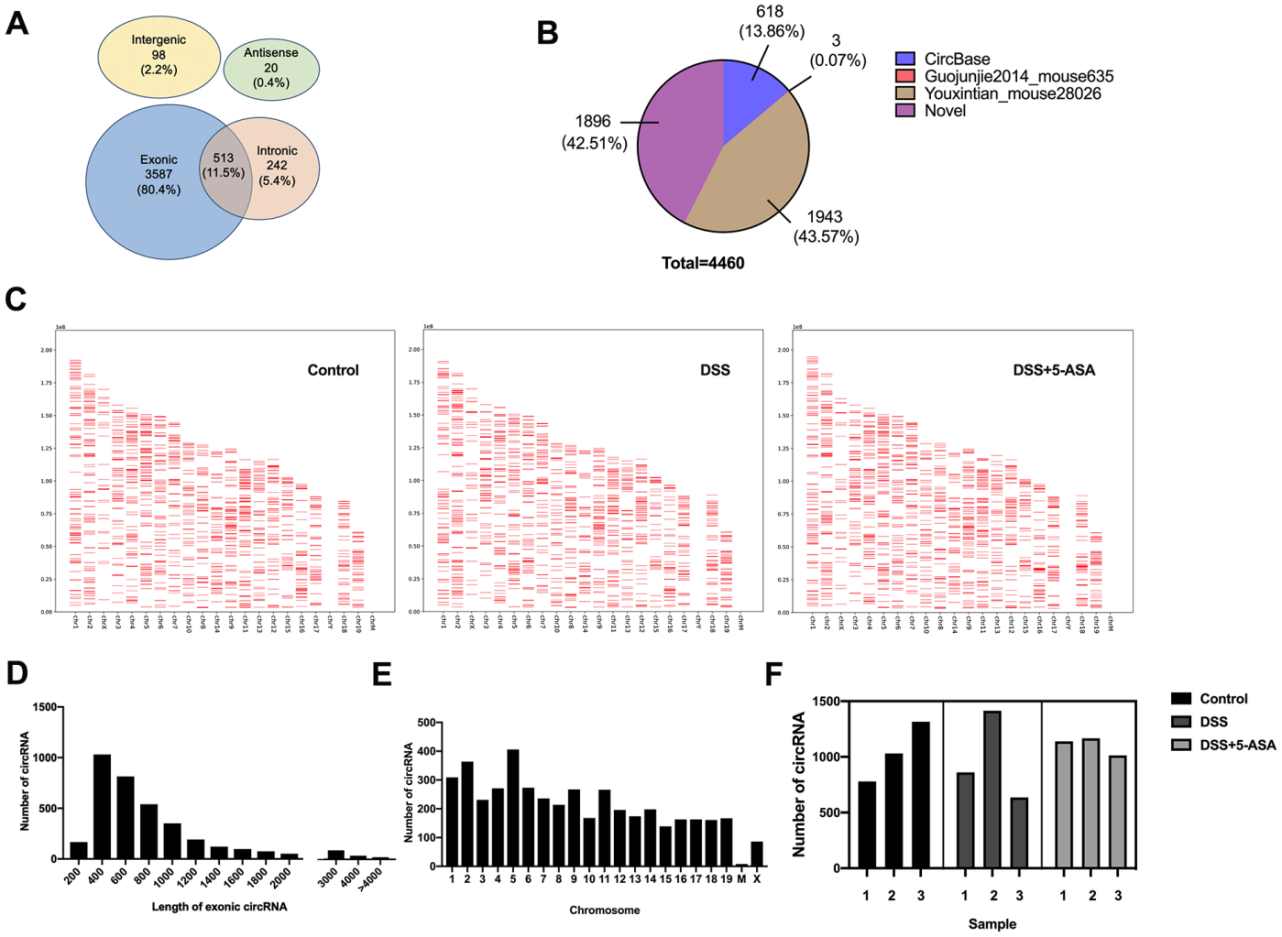
**Expression profiling of circRNAs in DSS-induced and mesalazine-treated IBD by RNA-seq.** (**A**) Venn plot was used to show the different genomic locations of the detected circRNAs. (**B**) Pie graph was conducted to exhibit the source of the detected circRNAs. (**C**) Positions and densities of the detected circRNAs. X-axis: distributed chromosome. Y-axis: the length of chromosome. The short red lines represent the position and density of these circRNA on the chromosome. (**D**) Length of all exonic circRNAs. X-axis: length of exonic circRNAs. Y-axis: the number of exonic circRNAs of different length. (**E**) Chromosomal distribution of the detected circRNAs. X-axis: distributed chromosome. Y-axis: the number of circRNAs of different chromosome. (**F**) The number of detected circRNAs in each group.

The RNA-seq analysis showed that a total of 4460 circRNAs were detected in mice colonic tissues from the control, DSS and DSS+5-ASA groups. Most of these circRNAs were transcribed from protein coding exons and others were from introns, intergenic or antisense (Figure 2A). Among these circRNAs, 2564 circRNAs were included in circBase and other published data, such as Guojunjie2014 [15], and 1896 circRNAs were labeled with novel (Figure 2B). As shown in Figure 2C–2E, most exon circRNAs in the three groups were distributed from chromosome 1 to 19, among which more were distributed on chromosome 1, 2 and 5, and the nucleotide length of these circRNAs was less than 1000. Figure 2F shows the numbers of circRNA in each group. A total of 3125 circRNAs were detected in the control group, 2910 in the DSS group and 3319 in the DSS + 5-ASA group.

**Figure 3:**
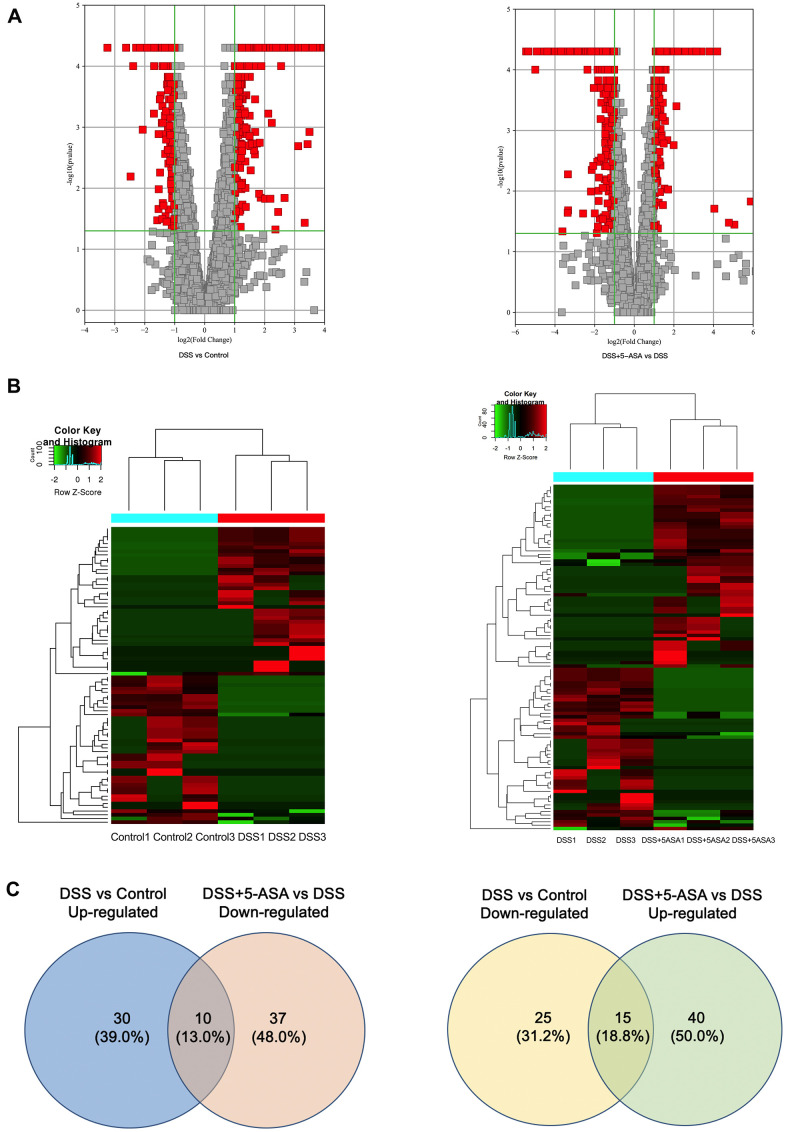
**Bioinformatic analysis of the detected circRNAs between DSS vs. control and DSS+5-ASA vs. DSS group.** (**A**) Volcano plot shows the significantly differentially expressed circRNAs in a visible way and the vertical green lines correspond to 2.0-fold up- and down-regulation and the horizontal line represents the 0.05-p-value. (**B**) Hierarchical clustering was used to evaluate the differentially expressed circRNAs when comparing with each sample of control, DSS and DSS+5-ASA group. Columns indicated the expression pattern of each sample; the green and red line represented the low and high expression level. (**C**) Venn plot shows the number of the dysregulated circRNAs in DSS vs. control and DSS+5-ASA vs. DSS group.

Among the 4460 circRNAs, we identified 182 differentially expressed circRNAs, which were displayed by volcano plot and heat map with fold change≥2.0 and P≤0.05 (Figure 3A, 3B). These significantly differentially expressed circRNAs included 40 up-regulated and 40 down-regulated circRNAs, respectively, when comparing the DSS with the control group, and 55 up-regulated and 47 down-regulated circRNAs, respectively, when comparing the DSS+5-ASA with the DSS group (Figure 3C). Among them, there were 12 overlapping circRNAs in (up-regulated) the DSS vs. control and (down-regulated) DSS + 5-ASA vs. DSS comparisons, and 16 in (down-regulated) the DSS vs. control and (up-regulated) DSS + 5-ASA vs. DSS comparisons. The detailed expression profiling of differentially expressed circRNAs and the full profiling of all circRNAs are provided in supplementary files (Supplementary Tables 1, 2, respectively).

### GO (gene ontology) and KEGG (kyoto encyclopedia of genes and genomes) analysis of DE circRNA-associated genes

**Figure 4:**
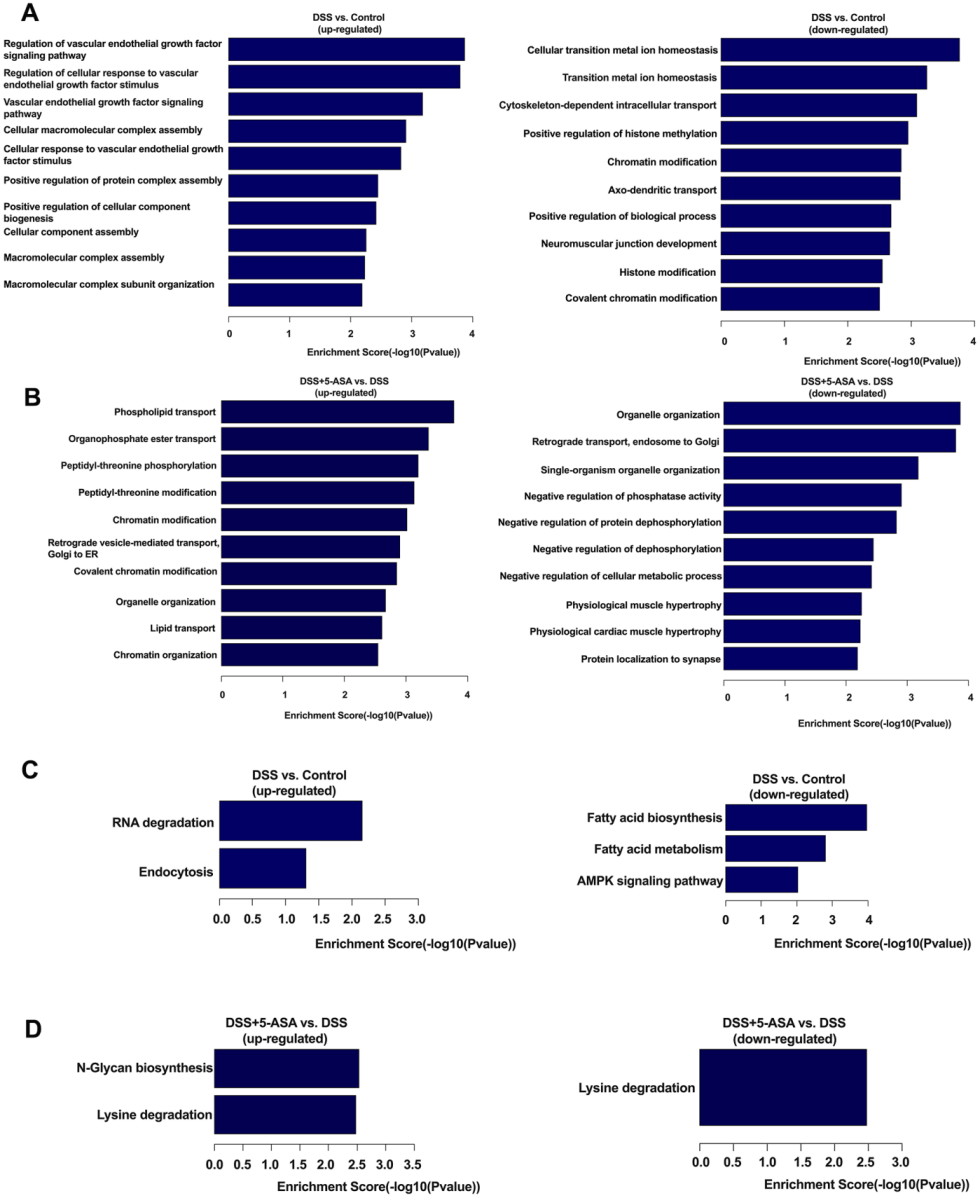
**GO and KEGG pathway analysis of differentially expressed circRNA-associated genes.** Top ten enriched BP terms in DSS vs. control (**A**) and DSS+5-ASA vs. DSS (**B**) group. All pathways in DSS vs. Control (**C**) and DSS+5-ASA vs. DSS (**D**) group.

When conducting GO analysis, the most enriched biological process (BP) terms of the DSS group were regulation of vascular endothelial growth factor signaling pathway (up-regulated, Figure 4A) and cellular transition metal ion homeostasis (down-regulated, Figure 4A) when comparing with the control group. Meanwhile, we found that when comparing the DSS+5-ASA with the DSS group, phospholipid transport (up-regulated, Figure 4B) and organelle organization (down-regulated, Figure 4B) were the most important BP terms.

In the cellular component (CC) domain, the most enriched terms were CCR4-NOT complex (up-regulated, Supplementary Figure 2A) and intracellular part (down-regulated, Supplementary Figure 2A) in the DSS group compared with the control group. Intracellular (up-regulated, Supplementary Figure 2B) and intracellular part (down-regulated, Supplementary Figure 2B) were the most enriched terms in the DSS+5-ASA compared with the DSS group.

As for the molecular function (MF) terms, metal ion binding (up-regulated, Supplementary Figure 3A) and enzyme activator activity (down-regulated, Supplementary Figure 3A) were the most important terms when conducting comparisons between the DSS and the control group. The most enriched MF terms were phospholipid transporter activity (up-regulated, Supplementary Figure 3B) and protein binding (down-regulated, Supplementary Figure 3B) in the DSS+5-ASA group compared with the DSS group. The complete results of the GO enrichment analysis are provided in Supplementary Table 3.

The results of KEGG analysis showed that a total of eight pathways were identified when conducting the two comparisons (DSS vs. control and DSS+5-ASA vs. DSS). These identical pathways were related to RNA degradation, endocytosis, fatty acid biosynthesis and metabolism, adenosine monophosphate-activated protein kinase (AMPK) signaling pathway, N-Glycan biosynthesis and lysine degradation (Figure 4C, 4D). The complete results of the KEGG enrichment analysis are provided in Supplementary Table 4.

### Validation of selected circRNAs from differential expression profiling

**Figure 5:**
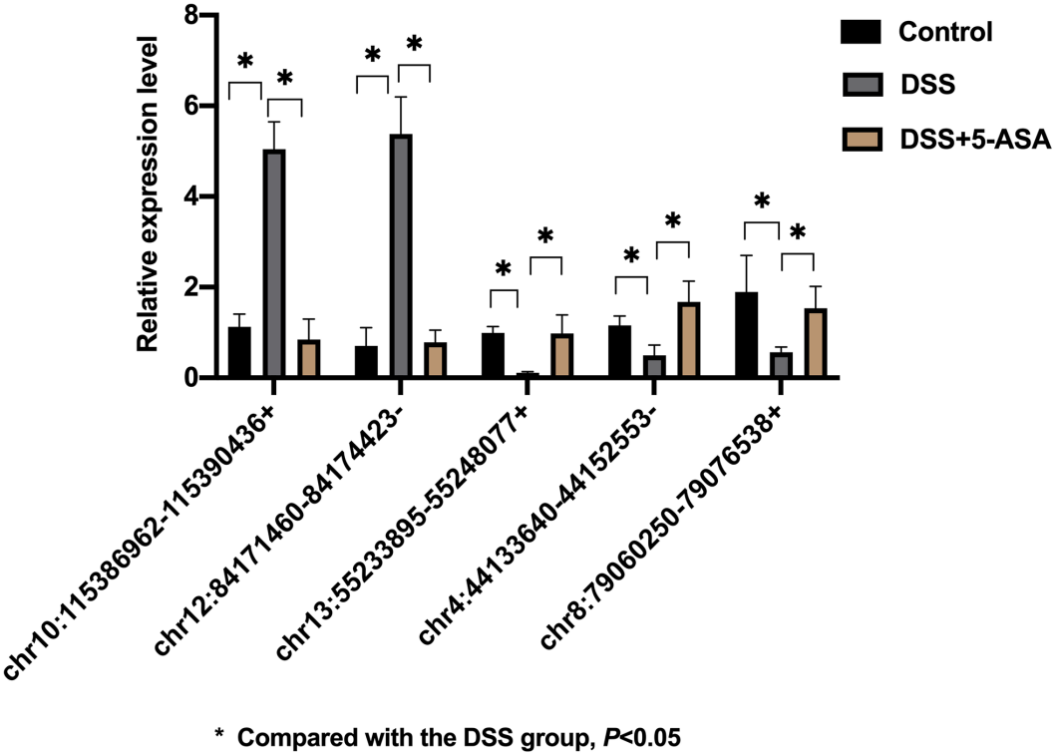
**qRT-PCR validation of selected circRNAs.** Five circRNAs were selected based on logFC and p-value for further validation by qRT-PCR. The result was consistent with the result of RNA-seq. *p < 0.05.

Five cicrRNAs were selected from the 182 differentially expressed circRNAs for validation based on logFC and p-value (Table 1), the sequences of primers of these circRNAs were listed in S1 Supplementary Table 5. As shown in Figure 5, chr10:115386962-115390436+ and chr12:84171460-84174423- were up-regulated, chr13:55233895-55248077+, chr4:44133640-44152553- and chr8:79060250-79076538+ were down-regulated in the DSS group when comparing with the control and the DSS+5-ASA group. The results of qRT-PCR were consistent with the RNA-seq results, highlighting the good reliability of the expression profiling.

**Table 1:** Five differentially expressed circRNAs were selected for validation by qRT-PCR.

**CircRNA name**	**Type**	**logFC^a^**	**P-value**	**Best transcripts**	**Gene name**
**up-regulated (DSS vs Control) and down-regulated (DSS+5-ASA vs DSS)**					
chr10:115386962-115390436+	exonic	4.786	0.002	NM_144910	Cnot6l
chr12:84171460-84174423-	exonic	4.056	0.017	NM_001081423	Ttll5
**down-regulated (DSS vs Control) and up-regulated (DSS+5-ASA vs DSS)**					
chr13:55233895-55248077+	exonic	4.912	0.002	NM_025909	Oma1
chr4:44133640-44152553-	exonic	4.380	0.010	NM_001081557	Camta1
chr8:79060250-79076538+	exonic	3.506	0.047	NM_027118	Cdk13

^a^FC, Fold Change.

### Prediction of circRNA-miRNA-mRNA network

The prediction network result showed a network of circRNA-miRNA consisting of 65 circRNAs and 183 miRNAs (Supplementary Figure 4).

**Figure 6:**
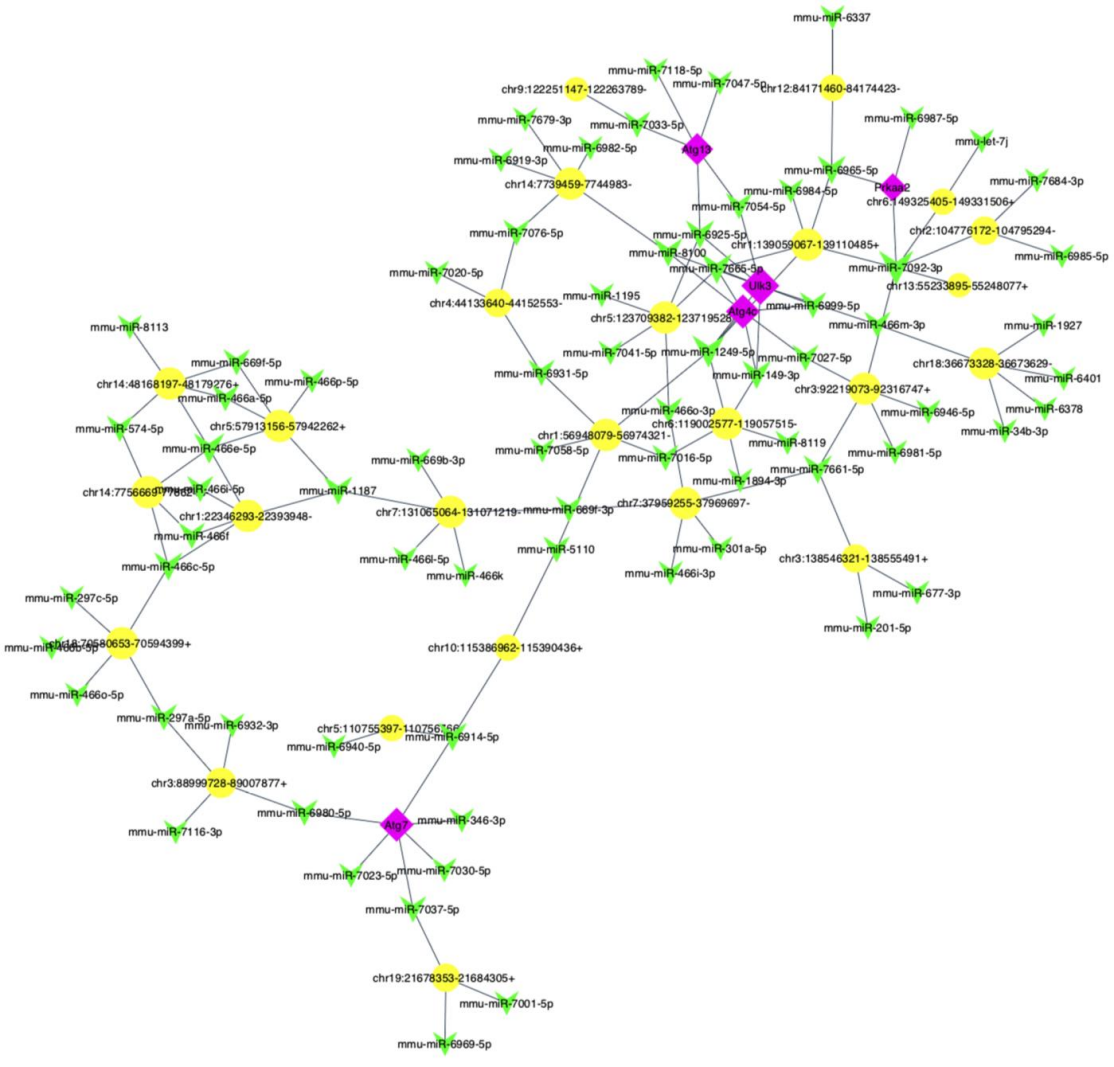
**The predicted autophagy-related circRNA-miRNA-mRNA network analysis.** The network consisted of 25 circRNAs (yellow nodes), 72 miRNAs (green nodes) and 5 mRNAs (purple nodes).

**Figure 7:**
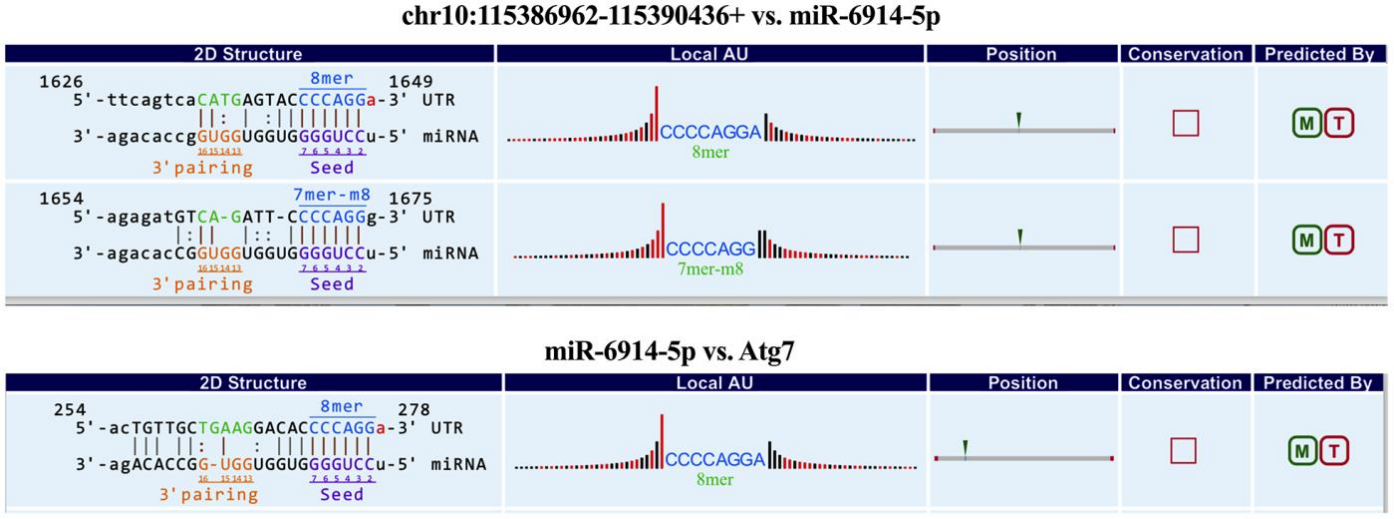
**The predicted binding sites of mmu-miR-6914-5p in chr10:115386962-115390436+ and Atg7.**

**Figure 8:**
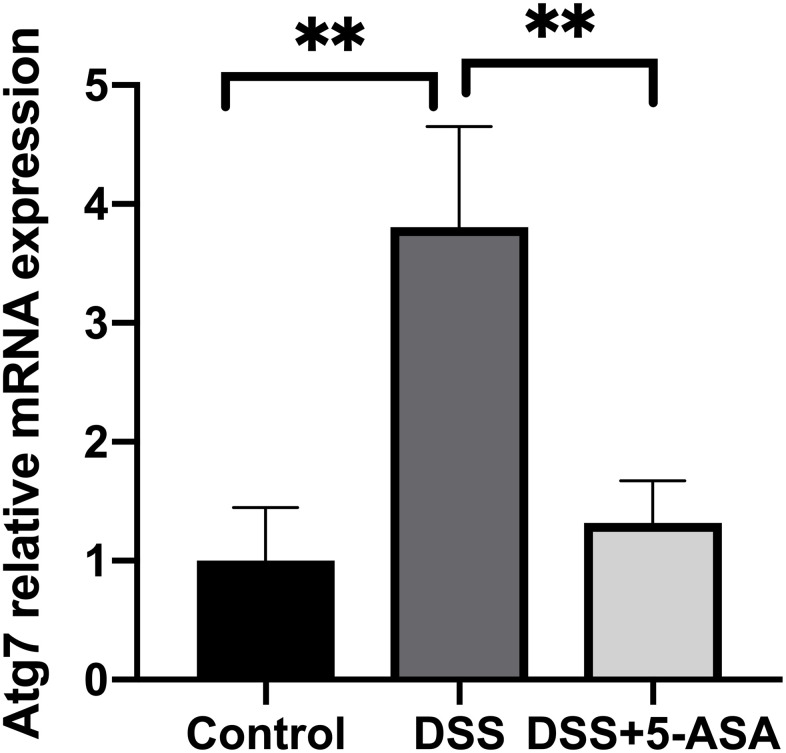
**qRT-PCR validation of selected mRNA.** qRT-PCR was performed to measure the expression level of Atg7. The trend of this mRNA was consistent with its corresponding circRNA. **p < 0.01.

Furthermore, to explore whether circRNAs acted as “miRNA sponges” [10] for executive function, we predicted the interactions of these differentially expressed circRNAs, miRNAs and associated mRNAs in some specific IBD-related pathways, such as JAK/STAT pathway. The results showed that the network of chr10:115386962-115390436+/mmu-miR-6914-5p/Atg7 (Autophagy related protein 7) seemed to be related to autophagy (Figure 6). Therefore, according to the prediction network and verified qRT-PCR results, chr10:115386962-115390436+ was selected to be further analyzed. The top five prediction miRNA binding sites were list in Supplementary Table 6. As the most likely target miRNA, the binding site of mmu-miR-6914-5p in chr10:115386962-115390436+ and the interaction of mmu-miR-6914-5p and Atg7 were shown in Figure 7. We performed qRT-PCR again to measure the expression level of Atg7 for validation, the primer sequence of the mRNA was list in Supplementary Table 7. The result suggested that this mRNA was significant amplified in the DSS group, which had a similar expression trend with its corresponding circRNA (Figure 8).

## DISCUSSION

To the best of our knowledge, this study is the first to explore the circRNAs expression profiling in colon tissues from mesalazine-treated IBD mice by performing next-generation RNA-seq. Based on the DSS-induced IBD mouse model that we successfully established, we applied RNA-seq to explore the circRNAs expression profiling in colon tissues that were randomly selected from the control, DSS and DSS+5-ASA groups. We found a number of differentially expressed circRNAs and related pathways (e.g., RNA degradation, endocytosis and AMPK signaling pathway), particularly an important circRNA-miRNA-mRNA pathway (chr10:115386962-115390436+/mmu-miR-6914-5p/Atg7) related to autophagy. Furthermore, we performed primary validation of the identified circRNAs. Our results provide a new understanding of the mechanism of IBD pathogenesis and mesalazine treatment.

The establishment and application of animal models, especially mouse models, are a bridge between basic research and clinical practice for disease research. Animal model is important for exploring molecular mechanisms of diseases, such as transplantation/induction tumor formation model for cancers [16], temporary/permanent left coronary artery (LCA) ligation for heart failure [17]. For IBD research, DSS-induced mouse model is the most widely used because of its ease of operation and similarities with human UC [18]. It has been demonstrated that Lypd8 could promote the segregation of flagellated microbiota and colonic epithelia by using DSS-induced intestinal inflammation in Lypd8(-/-) mice [19]. In this study, we not only successfully established DSS-induced IBD models, but also demonstrated the effectiveness of mesalazine in treating DSS-induced IBD. We found that administration with mesalazine helped relieve body weight loss and colon shortening, improved mucosal inflammation and reduced the production of serum IL-6 and TNF-α in mice with DSS-induced IBD. These findings are consistent with a previous study by Y. Li et al [20], in which the authors reported that mesalazine markedly improved clinical symptoms and mucosal healing, also inhibited the release of inflammatory cytokines in TNBS/ethanol-induced UC mice.

In this study, we detected 182 differentially expressed circRNAs that may include potential regulators and help, at least in part, explain the therapeutic mechanism of mesalazine for DSS-induced IBD. Together with the literature, we assume that mesalazine is most likely to treat IBD through playing anti-inflammatory role, e.g., blocking induction of nuclear factor kappa B(NF-κB) mediated by pro-inflammatory cytokines to enhance epithelial barrier function [21, 22]. Our findings showed that the most enriched BP terms when DSS+5-ASA group comparing with DSS group were almost related to inflammation response including vascular endothelial growth factor signaling pathway, phospholipid transport and organelle organization. These terms were associated with the activation of TNF-α and NF-κB pathway, indicating that mesalazine may downregulate the expression levels of certain pro-inflammation cytokines and pathways to play a protective role by changing the circRNA profiling of inflammatory colon tissue, which requires further validations from more studies. Our results strengthen some previous reports stating that the dysregulated of inflammation-related pathways lead to the abnormal inflammatory response in IBD [23].

It is widely recognized that mesalazine activates peroxisome proliferator-activated receptor (PPAR) gamma to attenuate colitis, which is closely associated with inhibition of NF-κB-dependent signaling. Results obtained in this study also suggest possible roles of circRNAs in the NF-κB-dependent pathway. Prior studies have suggested certain circRNAs may be involved in the regulation of NF-κB signaling pathway. For example, ssc_circ_009380 promoted activation of NF-κB pathway by binding miR-22 during Transmissible gastroenteritis virus (TGEV)-induced inflammation [24]; circUBAP2-mediated ceRNA network modulated pancreatic adenocarcinoma by regulating NF-κB signaling pathway and the function of immune cells [25]; circ_0075932 directly bound with the RNA-binding protein PUM2, which was reported to positively regulated AuroraA kinase, thus activating the NF-κB pathway to display a significantly promoting effect on inflammation and apoptosis in dermal keratinocytes [26]. Thus, we speculate that mesalazine may indirectly regulate PPAR-γ by influencing the profiling of certain circRNAs that can inhibit NF-κB pathway, so as to play a role in alleviating colonic inflammation.

Meanwhile, we found eight enriched pathways based on KEGG analysis, which were fatty acid biosynthesis and metabolism, AMPK signaling pathway, N-Glycan biosynthesis and lysine degradation, RNA degradation and endocytosis. Short chain fatty acids (SCFA), such as acetate and butyrate, which are metabolized by gut bacteria have been reported to mitigate inflammation in IBD animal models [27]. Recent findings showed that SCFAs, especially butyrate, were critical to strengthen gut barrier function and maintain intestinal homeostasis [28]. AMPK was involved in chronic inflammatory disorders as an important role. Some studies suggested AMPK regulated inflammatory responses of IBD through the inhibition of signaling pathways like NF-κB pathway [29, 30]. Moreover, AMPK was thought to activate autophagy via restraining certain signaling pathways (e.g. mTORC1 pathway) and directly phosphorylate some ATG proteins (e.g. ULK1, ATG9A, Beclin 1) [31, 32]. Metformin, a widely used drug for diabetes, may activate AMPK indirectly through mitochondrial depletion of ATP, and this activation further stimulates autophagy to treat diabetes [33]. Building on the findings above, we hypothesize that mesalazine may also indirectly play an effective role in regulating autophagy through AMPK. Listing more similar examples are possible but it is beyond the scope of this study. All these pathways can be tested when experiments are available, providing important clues for future research.

In addition, we predicted the circRNAs-miRNA-mRNA network in specific IBD-related pathways, including JAK/STAT and autophagy pathway. The results indicated the pathway of chr10:115386962-115390436+/miR-6914-5p/Atg7 was likely related to autophagy. As the novel circRNA, chr10:115386962-115390436+ was upregulated in colon tissues of mice in the DSS group when comparing with the DSS+5-ASA group. Some miRNAs have been identified to be involved in IBD-associated autophagy, such as miR-30C [34], miR-130a [35], miR-142-3p [36] and miR-106b [37]. These miRNAs inhibited the expression of autophagy-related genes, then contributed to autophagy dysfunction and promoted IBD development. Atg7 has been shown to be essential for autophagy. It played as a critical role in the formation of vesicle organelles (AVO) at the early stage of autophagy [38], and inhibited apoptosis by reducing p53-dependent transactivation of apoptosis promoting genes (e.g. Puma, Bax). Moreover, Atg7 interacted with acetylated FOXO1 to induce autophagy [39]*.* Previous studies have demonstrated the loss of Atg7 was related to increased intestinal inflammation [40]. Furthermore, mesalamine exerted its therapeutic effects of IBD based on regulating autophagy in the intestine. Prior literature has showed that mesalazine could repress the expression of IL-1β in neutrophils; thus, influence regulated in development and DNA damage responses 1 (REDD1)/autophagy pathway to alleviate inflammatory response in active UC [41]. Building on these findings above, it seems to be reasonable to assume that mesalazine may exert the anti-inflammatory effect through regulating Atg7-related autophagy progress. Although there are no literatures on functions of chr10:115386962-115390436+ and miR-6914-5p in IBD, we can infer that they may be associated with the development of IBD-related autophagy combining the preceding similar studies with our functional prediction experiments. Our study indicated that chr10:115386962-115390436+ may regulate the function and expression of Atg7 by serving as a miRNA sponge of miR-6914-5p.

Currently, a few literatures have reported the relationship between circRNA and IBD. Most of these studies selected peripheral blood from IBD patients and healthy individuals or inflamed and normal colorectal mucosa tissues from IBD patients as samples to perform RNA-seq and investigated the effect of circRNAs in IBD by comparing the different expression profiling of circRNA in IBD and normal group [12, 42]. To date, there is no study for circRNA sequencing of samples from mesalazine-treated IBD patients or animals. Therefore, this study provides a practical paradigm to explore molecular mechanisms of the treatment process of IBD (here we focused on mesalazine).

Limitations of this study included the small number of mice for each group, which should be increased in follow-up experiments to verify our findings. In addition, more experiments were needed to verify the hypothesis resulted from this study. For example, mesalazine might play a therapeutic role in IBD by regulating the NF-κB pathway. Therefore, it was necessary to further study the effects of mesalazine on NF-κB activation-related factors (e.g., COX-2, IL-1, p-NF-κB /P65), to improve the understanding of the therapeutic process of mesalazine for IBD.

In summary, our study was the first to present the circRNA expression profiling in colon tissues from mesalazine-treated IBD mice using RNA-seq. We suggested a number of potential IBD-related circRNAs and pathways, particularly highlighting an important circRNA-miRNA-mRNA pathway related to autophagy. These findings, although require further validations, provide new insights into the molecular mechanisms of IBD progression and clues for further research.

## MATERIALS AND METHODS

### DSS-induced and mesalazine-treated IBD model establishment

C57BL/6 mice (female, 10-week-old) were purchased from Shanghai Sippr-BK Laboratory Animal Co. Ltd (Shanghai, China). All mice were housed under conventional condition. Fifteen mice were randomly divided into three groups (five mice for each group): control, DSS, and DSS+5-ASA group after an acclimatization period. All experiments were approved by the Animal Experimentation Ethics Committee at Shanghai University of Traditional Chinese Medicine.

IBD was induced by administrated 2.5% DSS (36–50 kDa, MP Biomedicals, USA) in drinking water for seven days [43]. The mice of DSS+5-ASA group were received mesalamine (60 mg/kg [44] in phosphate-buffered saline (PBS), Ethypharm, France) by oral gavage twice a day for one week after IBD induction, and mice of control and DSS group were given the equal volume of PBS.

### Serum inflammation cytokines

To assess the inflammation severity, ELISA kits (pg/ml, Abcam, USA) were used to measure the expression levels of serum IL-6 and TNF-α.

### Total RNA isolation and quantification

Three colon tissue samples were selected randomly from each of the three groups. Total RNA was extracted using Trizol (TaKaRa, Japan) reagent. A NanoDrop ND-2000 instrument (Thermo Fisher Scientific, Waltham, MA, USA) was used to measure RNA quantification and quality. The OD260 / OD280 ratio was used as an indicator of RNA purity. The ratio is required to be close to 2.0 for pure RNA (a ratio between 1.8 and 2.1 is acceptable). RNA integrity and gDNA contamination were tested by denaturing agarose gel electrophoresis. The 28S and 18S rRNA bands is fairly sharp and intense, while the 5S rRNA bands is smaller and more diffuse. DNA contamination of the RNA preparation is evident as a high molecular weight smear or band migrating above the 28S rRNA band. Degradation of the RNA is reflected by smearing of rRNA bands.

### circRNA library construction and sequencing

High throughput whole transcriptome sequencing and subsequent bioinformatics analysis were all done by Cloud-Seq Biotech (Shanghai, China). In brief, rRNAs were removed from total RNA using Ribo-Zero rRNA Removal Kits (Illumina, USA) according to the manufacturer's instructions. Using rRNA-depleted RNAs with TruSeq Stranded Total RNA Library Prep Kit (Illumina, USA), we constructed the CircRNA library and then quantified it using the Bio Analyzer 2100 system (Agilent Technologies, USA). Quality control was performed for the library. A total of 10 pM libraries were denatured as single-stranded DNA molecules, captured on Illumina flow cells, amplified *in situ* as clusters and finally sequenced for 150 cycles on Illumina HiSeq Sequencer following the manufacturer’s instructions.

### qRT-PCR analysis

Total RNAs were reversely transcribed into cDNA by a Gene Amp PCR System 9700 (Applied Biosystems, USA) and qRT-PCR amplification was conducted in ViiA 7 Real-time PCR System (Applied Biosystems, USA). CircRNA expression level was defined as the comparative cycle threshold (Ct) and calculated by the double-standard curve method. Primers (Supplementary Table 5) used for qRT-PCR were provided by Yingjun Biotech (Shanghai, China). Three samples in each group were used for qRT-PCR verification.

### Bioinformatic analysis

Paired-end reads were harvested from Illumina HiSeq 4000 sequencer. After 3’ adaptor-trimming and low-quality reads removing by cutadapt software [45], the high-quality trimmed reads were used to analyze circRNAs. Chimeric read alignments can be reported by the fast and splice-aware read mapper STAR [46]. Only the reads over the back-splice junctions are typically used to detect and identify circRNA with DCC software [47] and annotated with circBase database [48]. More details can be found in a previous publication [47]. The edgeR software [49] was used to normalize the data and perform differentially expressed circRNA analysis. More specifically, the quasi-likelihood (QL) F-test was used to calculate the difference of circRNA expression between groups. We did two comparisons: DSS vs. control and DSS+5-ASA vs. DSS and presented the results separately. The differentially expressed circRNAs were identified with the following criteria: fold change≥2.0 and P≤0.05. GO and KEGG analysis were performed to investigate the biological functions of the differentially expressed circRNA-associated genes. GO analysis includes three domains: biological process (BP), cellular component (CC) and molecular function (MF). We presented the top ten (if there were) GO BP terms and KEGG pathways in the main text and included other results in supplementary materials.

### circRNA annotation

The circRNAs were divided into exon circRNA, intron circRNA or intergenic circRNA, etc., according to the alignment positions of the two ends of the circRNA. The identified circRNAs were then linked to the circBase database based on the genomic location of the circRNA for annotation. The newly identified circular RNAs were labeled with novel.

### circRNA-miRNA analysis

Cloudseq's home-made miRNA target prediction software based on miRanda and TargetScan [50, 51] and Cytoscape software [52] were applied to predict the potential relationship between the differentially expressed circRNAs and miRNAs and construct a circRNA–miRNA network, respectively.

### Statistical analysis

All results were reported as the mean ± standard deviation (SD). GraphPad Prism 6.0 was used to analyze the data. The Mann–Whitney test (one side) was used for comparisons of relative expression levels between the control and DSS groups, the DSS and DSS+5-ASA groups (qRT-PCR analysis). A P-value < 0.05 was considered statistically significant.

## Supplementary Material

Supplementary Figures

Supplementary Table 1

Supplementary Table 2

Supplementary Table 3

Supplementary Table 4

Supplementary Tables 5, 6, and 7
