# Supplementary material for: circRNA expression profiling of colon tissue from mesalazine-treated mouse of inflammatory bowel disease reveals an important circRNA-miRNA-mRNA pathway
Source: Aging (Albany NY). 2021 Mar 26;13(7):10187–207. doi: 10.18632/aging.202780 (PMC8064189; doi:10.18632/aging.202780)
Supplement: Supplementary Figures [file aging-13-202780-s001.pdf]

SUPPLEMENTARY FIGURES

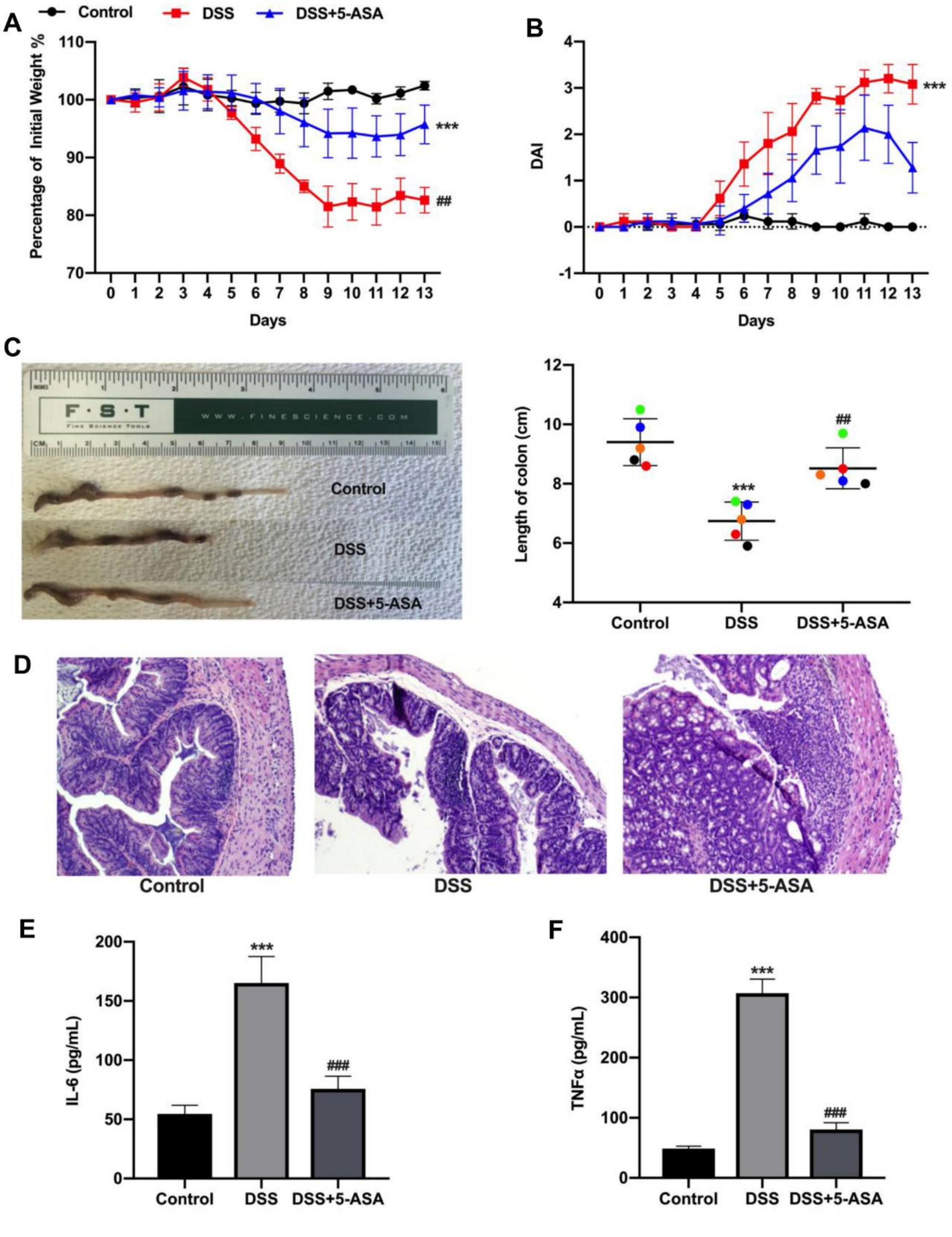

Supplementary Figure 1. Mesalazine attenuated DSS-induced IBD in mice. (**A**) Body weight, as a percent of initial weight, assessed daily. (**B**) DAI, the combined score of weight loss, stool consistency and bleeding, assessed daily. (**C**) Colon length measured at the last day of the experiment. (**D**) Representative H&E-stained distal colonic sections (magnification, x100). (**E**, **F**) The protein levels of inflammation-related cytokines IL-6 and TNF-α in plasma, determined by ELISA. Results are expressed as means ± SD; n = 5 mice per group. ***p < 0.001, vs. control group; ## p < 0.01, ### p < 0.001 vs. DSS group.

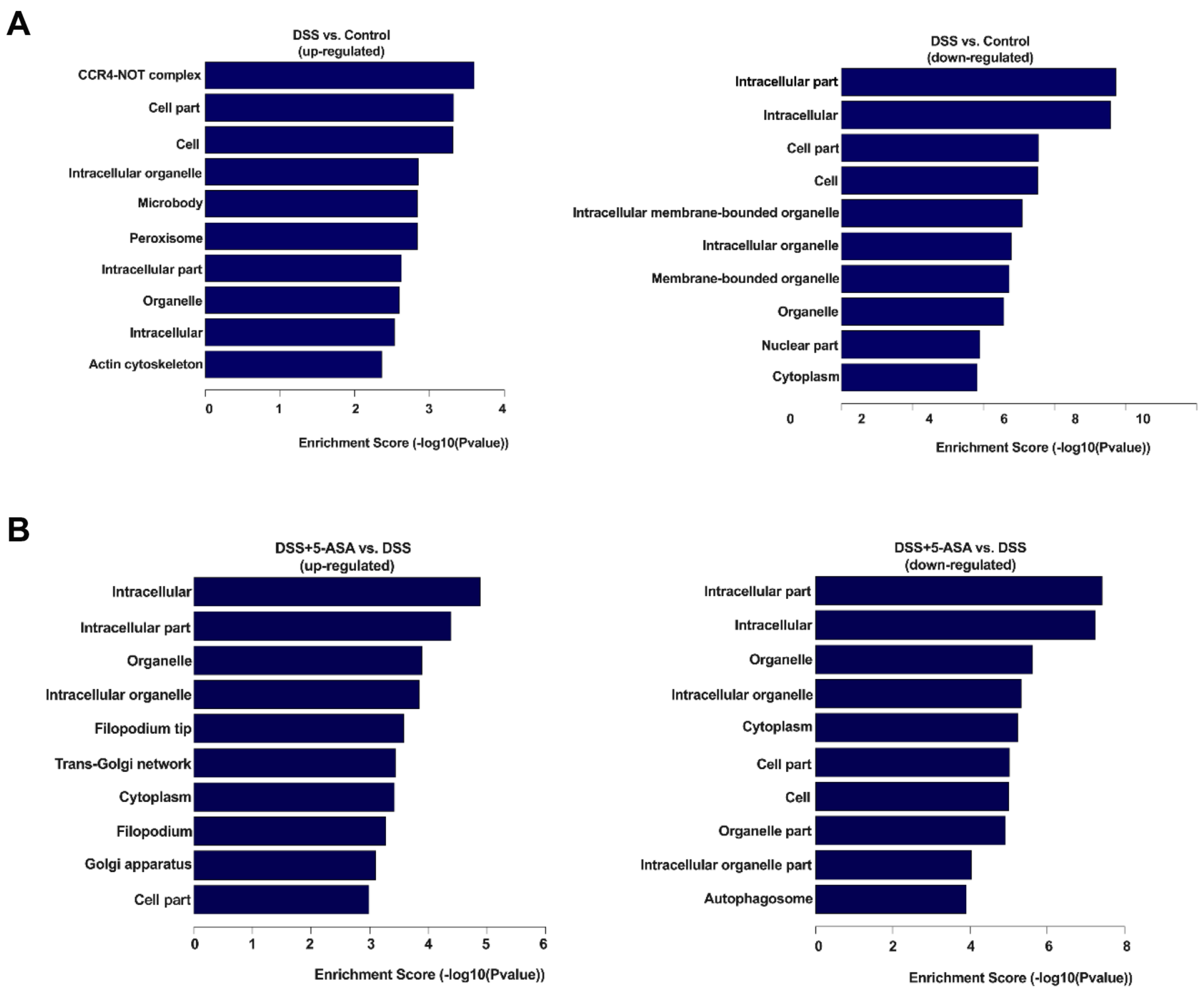

Supplementary Figure 2. CC analysis of differentially expressed circRNA-associated genes. Top ten enriched CC terms in DSS vs. control (**A**) and DSS+5-ASA vs. DSS (**B**) group.

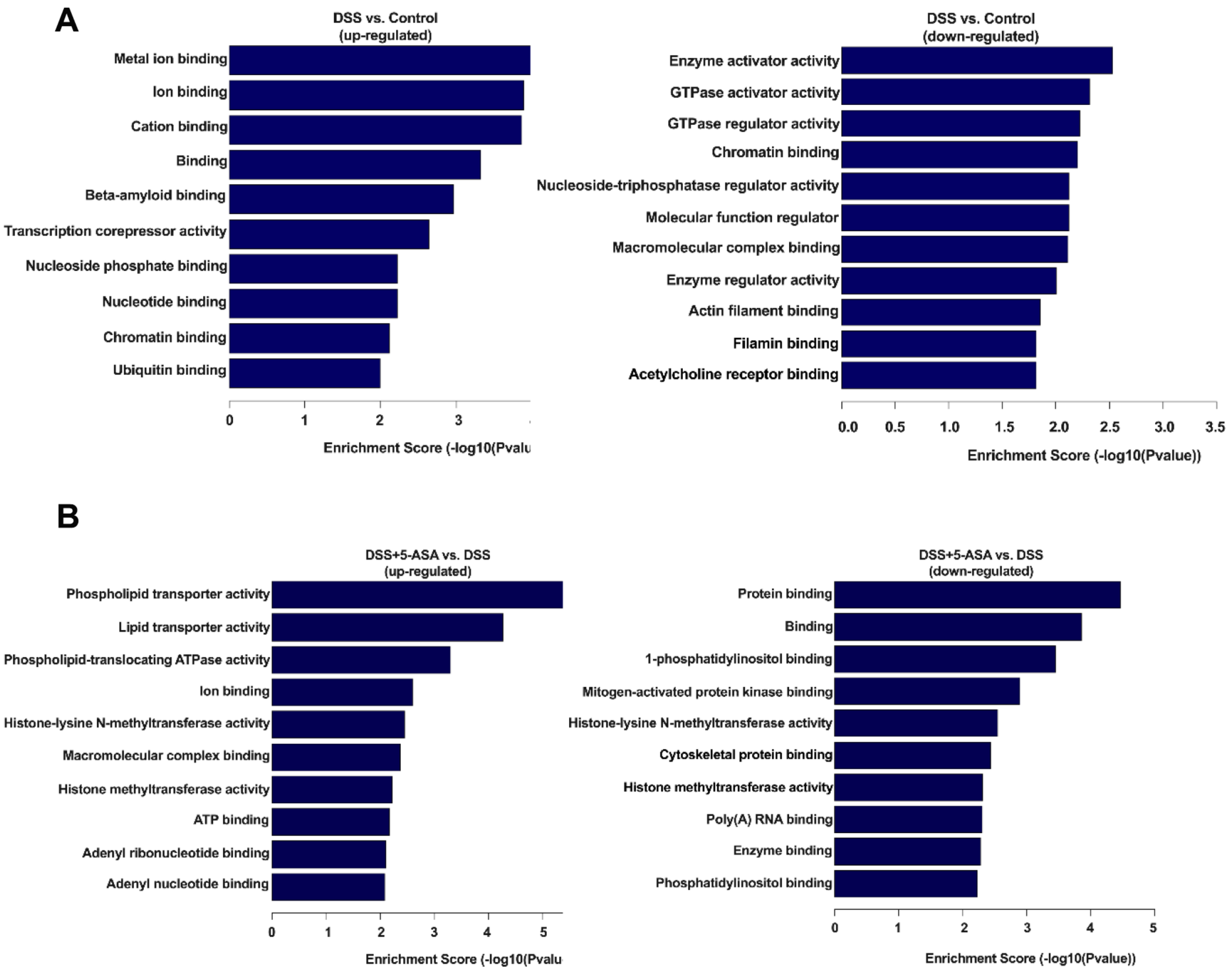

Supplementary Figure 3. MF analysis of differentially expressed circRNA-associated genes. Top ten enriched MF terms in DSS vs. control (**A**) and DSS+5-ASA vs. DSS (**B**) group.

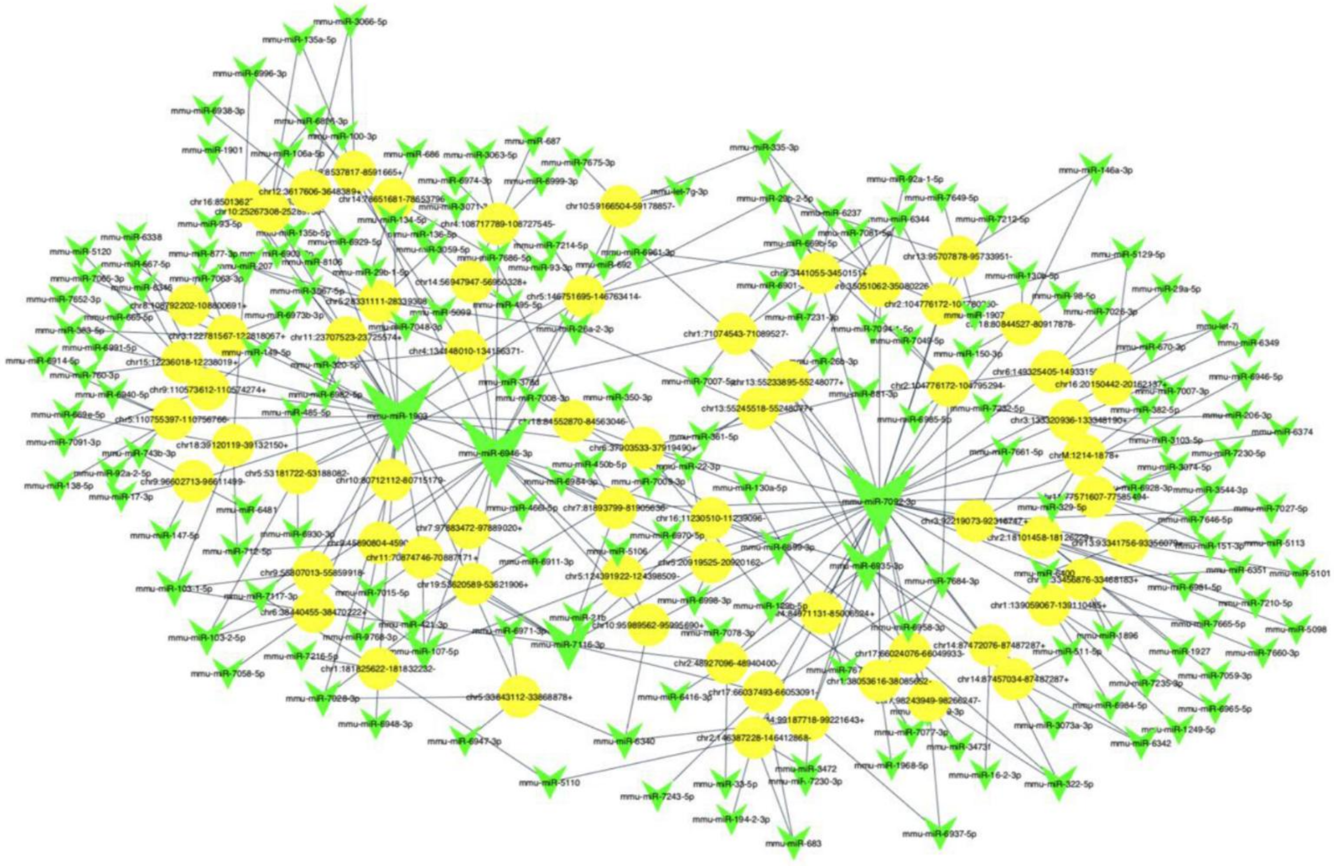

Supplementary Figure 4. The predicted circRNA-miRNA network analysis. The network consisted of 65 circRNAs (yellow nodes) and 183 miRNAs (green nodes).
