# Supplementary material for: circRNA expression profiling of colon tissue from mesalazine-treated mouse of inflammatory bowel disease reveals an important circRNA-miRNA-mRNA pathway
Source: Aging (Albany NY). 2021 Mar 26;13(7):10187–207. doi: 10.18632/aging.202780 (PMC8064189; doi:10.18632/aging.202780)
Supplement: Supplementary Tables 5, 6, and 7 [file aging-13-202780-s006.pdf]

SUPPLEMENTARY TABLES

Supplementary Table 5. Sequences of primers used for validation qRT-PCR of five selected circRNAs.

Gene	Primer name	Sequence	Tm	Product length
chr10:115386962-115390436+	1-Forward	CACTGGCTTTGGGTTTTTGT	60.01	218
	1-Reverse	TGAATGGCTCATGTTTGCAT	60.08	
chr12:84171460-84174423-	2-Forward	GAGGATGGGACTGGTCTTGA	60.05	202
	2-Reverse	CTAGGAAATGTCCGCTGGTC	59.69	
chr13:55233895-55248077+	3-Forward	CGGCTGAATGTTTGTGAGAA	59.84	180
	3-Reverse	CAAGGGCGTCTCTTGAATTT	59.31	
chr4:44133640-44152553-	4-Forward	CTCGGGGACTGACTAAAGCA	60.39	193
	4-Reverse	TGATGTCATCTCCCATGGTC	59.27	
chr8:79060250-79076538+	5-Forward	CCAACCACACAAAGCTGAAA	59.73	205
	5-Reverse	TAGGACGCTTCTGACGGAGT	60.01	
GAPDH	6-Forward	GGCCTCCAAGGAGTAAGACC	60.07	122
	6-Reverse	AGGGGAGATTCAGTGTGGTG	59.96	

Supplementary Table 6. Top five miRNA binding sites prediction of chr10:115386962-115390436+.

miRNA name	Number of binding site	Context+	Context	Structure	Energy
mmu-miR-6914-5p	2	-0.151	-0.184	585	-104.08
mmu-miR-677-3p	4	-0.397	-0.534	742	-95.96
mmu-miR-5110	1		-0.077	582	-104.58
mmu-miR-7064-5p	2	-0.083	-0.138	585	-67.12
mmu-miR-669b-5p	2	-0.506	-1.002	578	-53.27

Supplementary Table 7. Sequences of primers used for validation qRT-PCR of selected circRNA and mRNA.

Gene	Primer name	Sequence	Tm	Product length
chr10:115386962-115390436+	Mmu-CIR408-218F	CACTGGCTTTGGGTTTTTGT	60.01	218
	Mmu-CIR408-218R	TGAATGGCTCATGTTTGCAT	60.08	
Atg7	Mmu-Atg7-202F	TTGTAGCACCTGCTGACCTG	60.05	202
	Mmu-Atg7-202R	TGCAGGACAGAGACCATCAG	59.98	
GAPDH	Mmu-GAPDH-122F	GGCCTCCAAGGAGTAAGACC	60.07	122
	Mmu-GAPDH-122R	AGGGGAGATTCAGTGTGGTG	59.96	
